# Integrated transcriptomic and metabolomic analyses reveal the mechanisms underlying bio-organic fertilizer-mediated growth and nutrient enhancement in *Schisandra chinensis* (Turcz.) Baill

**DOI:** 10.3389/fpls.2025.1662470

**Published:** 2025-11-18

**Authors:** Ruiting Xu, Wenzhen Zhang, Sihui Chen, Simeng Song, Juane Dong, Zhi Yang, Yatuan Ma

**Affiliations:** 1 College of Chemistry and Pharmacy, Northwest A&F University, Yangling, Shaanxi, China; 2 Department of Ecological Environmental Engineering, Yangling Vocational and Technical College, Yangling, Shaanxi, China; 3 College of Life Science, Northwest A&F University, Yangling, Shaanxi, China

**Keywords:** *Schisandra chinensis*, bio-organic fertilizer, plant growth, nutrient content, transcriptomics, metabolomics

## Abstract

*Schisandra chinensis* (Turcz.) Baill. is a valuable and widely used traditional Chinese medicine with broad applications. As societal demand grows, enhancing both the yield and quality of this herb has become increasingly important, as these factors directly influence its clinical efficacy. Previous studies have indicated that bio-organic fertilizer can improve the yield and quality of *S. chinensis*. However, the underlying mechanisms regulating plant growth and nutrient accumulation remain poorly understood. In this study, we investigated the effects of different application rates of bio-organic fertilizer on the growth and nutrient content of *S. chinensis*. Using transcriptomic and metabolomic approaches, we elucidated the mechanisms by which bio-organic fertilization promotes plant growth and nutrient synthesis. The results demonstrated that the application of bio-organic fertilizer significantly improved the fruits’ morphological traits and yield. Additionally, the contents of beneficial compounds such as lignans, flavonoids, polysaccharides, and polyphenols were elevated. Among all treatments, the application of 100 kg/667 m² bio-organic fertilizer resulted in the highest yield and quality of *S. chinensis*. Transcriptomic and metabolomic analyses further revealed that bio-organic fertilizer enhances plant growth and nutrient metabolism, consistent with the observed phenotypic improvements. Taken together, this study employs a multi-omics approach to uncover the phenotypic and molecular responses of *S. chinensis* to bio-organic fertilization. The identified differentially expressed genes and accumulated metabolites provide new insights into the mechanisms by which bio-organic fertilizer improves the quality and yield of *S. chinensis*.

## Introduction

*Schisandra chinensis* (Turcz.) Baill., a member of the *Magnoliaceae* family, is a valuable traditional Chinese medicine (TCM) derived from dried mature fruits. The medicinal efficacy of *S. chinensis* is largely attributed to the bioactive compounds present in these fruits, which are crucial for its clinical applications. With growing demand over time for both the quantity and quality of this herb, supply constraints have become evident (Liu et al., 2018; Huang et al., 2019). However, little was known about how bio-organic fertilizer regulates *S. chinensis* growth and nutrient accumulation at the molecular level.

Wild resources are limited and insufficient to meet the substantial market needs. Moreover, the domestication and cultivation of *S. chinensis* started relatively late, and agronomic techniques remain underdeveloped. As a result, both the yield and quality of cultivated *S. chinensis* currently fall short of expectations.

The role of fertilizers in promoting plant growth and enhancing nutrient uptake has been a topic of considerable interest across numerous studies (Titulaer and Slangen, 1990; Rahman et al., 2011; Hashemabadi et al., 2018; Nikolaou et al., 2023; Yarsi, 2023)—for instance, research has demonstrated the effects of fertilization on maize seedling growth and foliar nutrient content (Hu et al., 2008) as well as improvements in plant growth and sugar yield in sugar maple through fertilization, underscoring the value of foliar and sap analysis in assessing nutritional requirements (Leech and Kim, 1990). Other works have revealed the long-term growth responses of ponderosa pine seedlings to controlled-release fertilizers (Fan et al., 2002) and examined how fertilizer application moderates the effects of simulated root herbivory on growth and biomass allocation in *Solidago canadensis* (Schmid et al., 1990). Studies also include the cost-effective cultivation of *Spirulina platensis* using NPK fertilizers (Singh et al., 2015), optimization of teak seedling growth through organic fertilization and soil medium manipulation (Séhouéto et al., 2015), and the impact of organic and inorganic fertilizers on NPK uptake and yield in sweet corn (Marlina et al., 2017). Additionally, fermented liquid fertilizers have been shown to promote growth in red pepper and tomato seedlings (Islam et al., 2013), while slow-release nitrogen fertilizers were found to enhance corn growth and yield in sandy soils (Wang et al., 2024). Collectively, these studies deepen our understanding of how fertilization strategies improve plant growth and nutrient acquisition across diverse species and conditions. A deeper understanding of the molecular mechanisms through which fertilizers promote plant growth and nutrient uptake is needed to develop more efficient cultivation strategies.

In recent years, multi-omics approaches have been widely applied to study plant responses to changing environments—for example, melatonin was found to alter the secondary metabolite profile of grape berry skin by promoting *VvMYB14*-mediated ethylene biosynthesis (Ma et al., 2021). Multi-omics analysis revealed cell aggregation as a defense strategy against perchlorate stress in *Chlamydomonas reinhardtii* (Zhang et al., 2023). Transcriptomic and metabolomic approaches demonstrated that autotoxic ginsenoside stress induces changes in root exudates, facilitating the recruitment of the beneficial *Burkholderia* strain B36 (Zhang et al., 2023). Integrated transcriptomic and metabolomic analyses have identified a protein module associated with preharvest apple peel browning (Wang et al., 2023), while further studies elucidated the resistance index and browning mechanisms of apple peel under high-temperature stress (Wang et al., 2024). A transcriptome and metabolome atlas revealed the contributions of sphingosine and chlorogenic acid to cold tolerance in *Citrus* (Xiao et al., 2024). Therefore, multi-omics has proven to be an effective strategy to investigate plant responses to environmental changes.

Recent research on the cultivation of Chinese medicinal herbs has advanced significantly, with a strong emphasis on sustainable development, quality control, and application potential. Progress is particularly nota ble in key species such as *Dendrobium officinale*. As highlighted by Hexigeduleng Bao, substantial advances have been made in the industrialization and seedling cultivation of this medicinal orchid, addressing key cultivation challenges to enhance its pharmacological benefits (Bao et al., 2024). These developments in propagation and cultivation techniques are central to broader efforts aimed at improving the yield and quality of medicinal plants.

As a valuable and widely used traditional Chinese medicine, *S. chinensis* has been shown in numerous studies to benefit from bio-organic fertilizer, which promotes its growth and enhances nutrient acquisition (Ma et al., 2012; Park and Lee, 2021). However, the optimal application rate of bio-organic fertilizer and the molecular mechanisms underlying its growth-promoting effects remain poorly understood. In this study, we investigated the phenotypic responses of *S. chinensis* to different levels of bio-organic fertilizer, particularly in terms of growth and nutrient composition. Additionally, we conducted transcriptomic and metabolomic analyses to elucidate the associated regulatory mechanisms. The results demonstrated that the application of bio-organic fertilizer improved fruit morphology and yield. Transcriptomic and metabolomic profiling further confirmed that bio-organic fertilizer enhances plant growth and nutrient accumulation, consistent with the observed phenotypic changes. The identified differentially expressed genes and differentially accumulated metabolites provide new insights into how bio-organic fertilizer promotes the growth and nutrient quality of *S. chinensis*.

## Materials and methods

### Plant materials and growth conditions

To investigate the effects of fertilizer on the growth and nutrient accumulation of *S. chinensis*, uniformly aged plants were cultivated in a single location under consistent climatic and environmental conditions. The experiment was conducted in July 2021 in Zhashui County, Shaanxi Province (33°46′ N, 109°19′ E; altitude, 1,276.7 m). The variety of *S. chinensis* used in this study has been described previously (He et al., 2024). The soil type was sandy loam with a pH of 7.9, containing 9.68 g/kg organic matter, 91.8 mg/kg available nitrogen, 25.49 mg/kg available phosphorus, 95.1 mg/kg available potassium, 8.16 mg/kg available iron, 9.19 mg/kg available manganese, 1.91 mg/kg available copper, and 1.32 mg/kg available zinc (**Table 1**). This study investigated the growth and quality of 3-year-old *S. chinensis* under reduced chemical fertilizer input combined with biodynamic organic fertilizer application. The experiment comprised six treatments, each replicated three times and randomly arranged in the field, resulting in a total of 18 plots containing 50 plants each. The soil was amended with different levels of fertilizer as follows: T0 (TK, control group, no fertilizer added); T1 (OM50), receiving 50 kg/667 m² bio-organic fertilizer, 35 kg/667 m² of 13-8–9 organic–inorganic compound fertilizer, 35 kg/667 m² of 15-6–24 compound fertilizer, and 17.5 kg/667 m² of 8-5–38 compound fertilizer; T2 (OM100), amended with 100 kg/667 m² bio-organic fertilizer (distinct from T1); and T3, amended with 150 kg/667 m² bio-organic fertilizer (**Table 2**) (Naher et al., 2021). The experiment was initiated in March 2023. On March 19, a long trench approximately 20 cm in width and depth was dug about 30 cm away from the dried roots of the *S. chinensis* plants. The biodynamic fertilizer and the organic–inorganic compound fertilizer specified in the experimental treatment were applied as base fertilizer in a single application. The remaining fertilizers were applied in late June. From March to August 2023, the growth and development of the schisandra plants were monitored. During this period, cultivation, weeding, and watering were performed as needed, and disease and pest control measures were implemented promptly. Sampling and measurements were conducted at different color-changing stages and at maturity. The plants were grown under these fertilization regimes through the flowering and fruiting stages. Mature fruits of *S. chinensis* were subsequently collected for phenotypic measurements, transcriptome sequencing, and metabolomic analysis.

**Table 1:** Soil nutrient overview at the *Schisandra chinensis* experimental site.

Soil type	pH value	Organic matter (g/kg)	Available nitrogen (mg/kg)	Available phosphorus (mg/kg)	Available potassium (mg/kg)	Available Fe (mg/kg)	Available Mn (mg/kg)	Available Cu (mg/kg)	Available Zn (mg/kg)
Sandy loam	7.9	9.68	91.8	25.49	95.1	8.16	9.19	1.91	1.32

**Table 2:** Experimental design for the fertilization of 3-year-old *Schisandra chinensis*.

Numbers	Treatments	Fertilization amount (kg/667 m^2^)			
		Bio-organic fertilizer	13-8-9 organic–inorganic compound fertilizer	15-6-24 compound fertilizer	8-5-38 compound fertilizer
T0	TK	0	0	0	0.0
T1	OM50	50	35	35	17.5
T2	OM100	100	35	35	17.5
T3	OM150	150	35	35	17.5

### Quantization of fruit production and quality

To investigate the effects of fertilizer on the growth and nutrient composition of *S. chinensis*, fruits from plants subjected to different fertilizer treatments were collected. The fruit shape index was measured, and fruit yield was quantified.

The total polyphenol content was determined using liquid chromatography–mass spectrometry (LC–MS), with a standard curve of *y* = 0.104*x* + 0.0604 (*R*² = 0.9993) (Mocan et al., 2014a). The total polysaccharide content was assessed via the phenol–sulfuric acid method, yielding a standard curve of *y* = 0.0062*x* – 0.0199 (*R*² = 0.9993). The total flavonoid content was measured using the NaNO_2_–Al(NO_3_)_3_ colorimetric method, with a standard curve of *y* = 1.2155*x* – 0.0103 (*R*² = 0.9991) (Zhao et al., 2022). Lignan content was analyzed using a Waters Alliance high-performance liquid chromatography (HPLC) system.

### RNA extraction, library preparation, and sequencing

To investigate the gene expression levels in *S. chinensis* under different fertilizer conditions, mature fruits were collected from both control and treatment groups for RNA extraction. All collected samples were immediately stored at –80°C. Total RNA was extracted using the EZNA Plant RNA Kit (R6827-01; Omega Bio-tek, USA).

The concentration of RNA was quantified with a Qubit^®^ 3.0 fluorometer, and integrity was assessed by 1% agarose gel electrophoresis. Samples passing quality control were used to construct RNA-Seq libraries. Methods for library preparation, RNA quality/quantity validation, and identification of differentially expressed genes (DEGs) were performed as previously described (Yu et al., 2024).

### Transcriptome analysis

In the sequencing data, differentially expressed genes (DEGs) were identified and functionally characterized. Comprehensive Gene Ontology (GO) enrichment and Kyoto Encyclopedia of Genes and Genomes (KEGG) pathway analyses of the DEGs were performed using the clusterProfiler R package. A stringent significance threshold of *p ≤*0.05 was applied to ensure that only statistically significant genes were selected for further analysis.

To gain deeper functional insights into the metabolic attributes associated with the DEGs, GO enrichment analysis was systematically conducted for both upregulated and downregulated genes across the three major categories: biological processes (BP), cellular components (CC), and molecular functions (MF). Additionally, the KEGG database was employed to elucidate key pathways and biological processes affected by the identified DEGs.

### Metabolome extraction and analysis

Metabolome extraction and analysis were carried out by Metware Biotechnology Co., Ltd. (Wuhan, China; http://www.metware.cn/) using a non-targeted metabolomic approach. Sample extracts were analyzed on a UPLC-ESI-MS/MS system (UPLC: Shim-pack UFLC SHIMADZU CBM30A; MS: Applied Biosystems 4500 Q TRAP).

Briefly, freeze-dried fruits of *S. chinensis* were ground to a fine powder in a mixer mill (MM 400, Retsch) with zirconia beads at 30 Hz for 1.5 min. Then, 0.1 g of the powder was weighed and extracted with 0.6 mL of 70% (v/v) aqueous methanol at 4°C overnight. After centrifugation at 10,000 × *g* for 10 min, the supernatant was subjected to solid-phase extraction using a CNWBOND Carbon-GCB SPE Cartridge (250 mg, 3 mL; ANPEL, Shanghai, China) and filtered through a 0.22-μm membrane (SCAA-104; ANPEL, Shanghai, China).

Data acquisition and validation were performed using Analyst software (v1.6.3; AB Sciex). Metabolite identification was conducted based on the Metware Database (MWDB), and quantification was achieved by integrating metabolite peak areas.

Differentially accumulated metabolites (DAMs) were defined as those with a variable importance in projection (VIP) value ≥1 and an absolute log_2_(fold change) ≥1.

### Transcriptomic and metabolomic variation and their association analysis

To investigate the associations between transcriptomic and metabolomic variations, pathways associated with DAMs were first retrieved from the KEGG database, along with the genes involved in these pathways. Subsequently, DEGs were identified through transcriptomic analysis. Finally, correlation coefficients between the DEGs and DAMs were calculated. Both the DEGs and their correlated DAMs were further analyzed within the context of the enriched KEGG pathways.

### Statistical analysis

In this study, all experimental data were obtained from three biological replicates and are presented as means ± standard deviation (SD). Biochemical and physiological data were analyzed using one-way analysis of variance (ANOVA), with a significance threshold set at *P <*0.05. Statistical analyses were performed using IBM SPSS Statistics v26.0.0. For comparisons between two groups, Student’s *t*-test was applied (http://www.physics.csbsju.edu/stats/), with significance levels defined as *p <*0.05 and *p <*0.01. Data visualization was carried out using Origin 2021. Transcriptomic and metabolomic analyses were conducted on the Majorbio Cloud Platform (www.majorbio.com). Cluster heatmaps were generated with TBtools (Chen et al., 2020), and correlations between transcriptomic and metabolomic data were assessed using two-way orthogonal partial least squares (O2PLS) analysis and Pearson correlation algorithms (Meng et al., 2022).

## Results

### Bio-organic fertilizer enhances *S. chinensis* growth and nutrient accumulation

**Figure 1:**
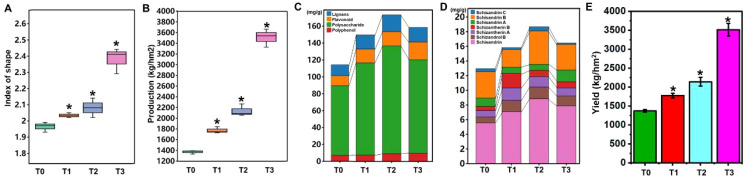
Effects of fertilization on the yield and quality of *S. chinensis*. **(A)** Fruit shape index under different fertilization conditions. **(B)** Fruit yield following fertilizer application. **(C)** Changes in the contents of lignans, flavonoids, polysaccharides, and polyphenols after fertilizer application. **(D)** Comparative analysis of lignan compounds, schisandrin C, schisandrin B, schisandrin A, schisantherin B, schisantherin A, schizandrol B, and schisandrin, in *S. chinensis*. **(E)** Yield of *S. chinensis* under different fertilization conditions. *P < 0.05, Student’s *t*-test.

As a well-known valuable traditional Chinese medicine, *S. chinensis* has been shown to exhibit improved yield and quality when cultivated in soil amended with bio-organic fertilizer. However, the specific mechanisms underlying its growth and nutrient accumulation remain poorly understood. To address this knowledge gap, we designed experiments to investigate the effects of bio-organic fertilizer on fruit development and nutrient composition in *S. chinensis*. The study was conducted in a dedicated experimental site where the native soil was identified as sandy loam with a pH of approximately 7.9 (**Table 1**). Based on preliminary soil nutrient analysis, four fertilization treatments were established: T0 (control, no fertilizer); T1 (50 kg/667 m² bio-organic fertilizer, 35 kg/667 m² of 13-8–9 organic–inorganic compound fertilizer, 35 kg/667 m² of 15-6–24 compound fertilizer, and 17.5 kg/667 m² of 8-5–38 compound fertilizer); T2 (T1 + 100 kg/667 m² bio-organic fertilizer); and T3 (150 kg/667 m² bio-organic fertilizer, applied differently from T1) (**Table 2**). Uniform-aged seedlings of *S. chinensis* were transplanted into the prepared soils. At fruit maturity, yield and quality parameters were measured. As illustrated in **Figures 1A, B**, both the fruit shape index and fruit production significantly increased in response to bio-organic fertilizer application. Among all treatments, the best group of soil in which the *S. chinensis* fruit shape index and fruit production were greatest was in T3.

Subsequently, we analyzed the nutrient composition of *S. chinensis* fruits. As shown in **Figure 1D**, the contents of lignans, flavonoids, polysaccharides, and polyphenols were significantly altered following the application of bio-organic fertilizer. Among all treatment groups, T2 resulted in the highest overall nutrient enrichment in the fruits.

Lignans, which are critical bioactive constituents in traditional Chinese medicine, include schisandrin C, schisandrin B, schisandrin A, schisantherin B, schisantherin A, schizandrol B, and schisandrin. Quantitative analysis revealed that schisandrin was the most abundant lignan component. Interestingly, the highest lignan content was observed in group T2, rather than T3. This suggests that while T3 promoted plant growth effectively, it did not optimally support the accumulation of nutrients, particularly lignans, in *S. chinensis*. These results demonstrate that fertilization strategies significantly influence both the yield and quality of *S. chinensis*. The optimal treatment for balanced growth and nutrient accumulation was determined to be T2.

### Transcriptome sequencing of *S. chinensis* under different bio-organic fertilizer treatments

**Figure 2:**
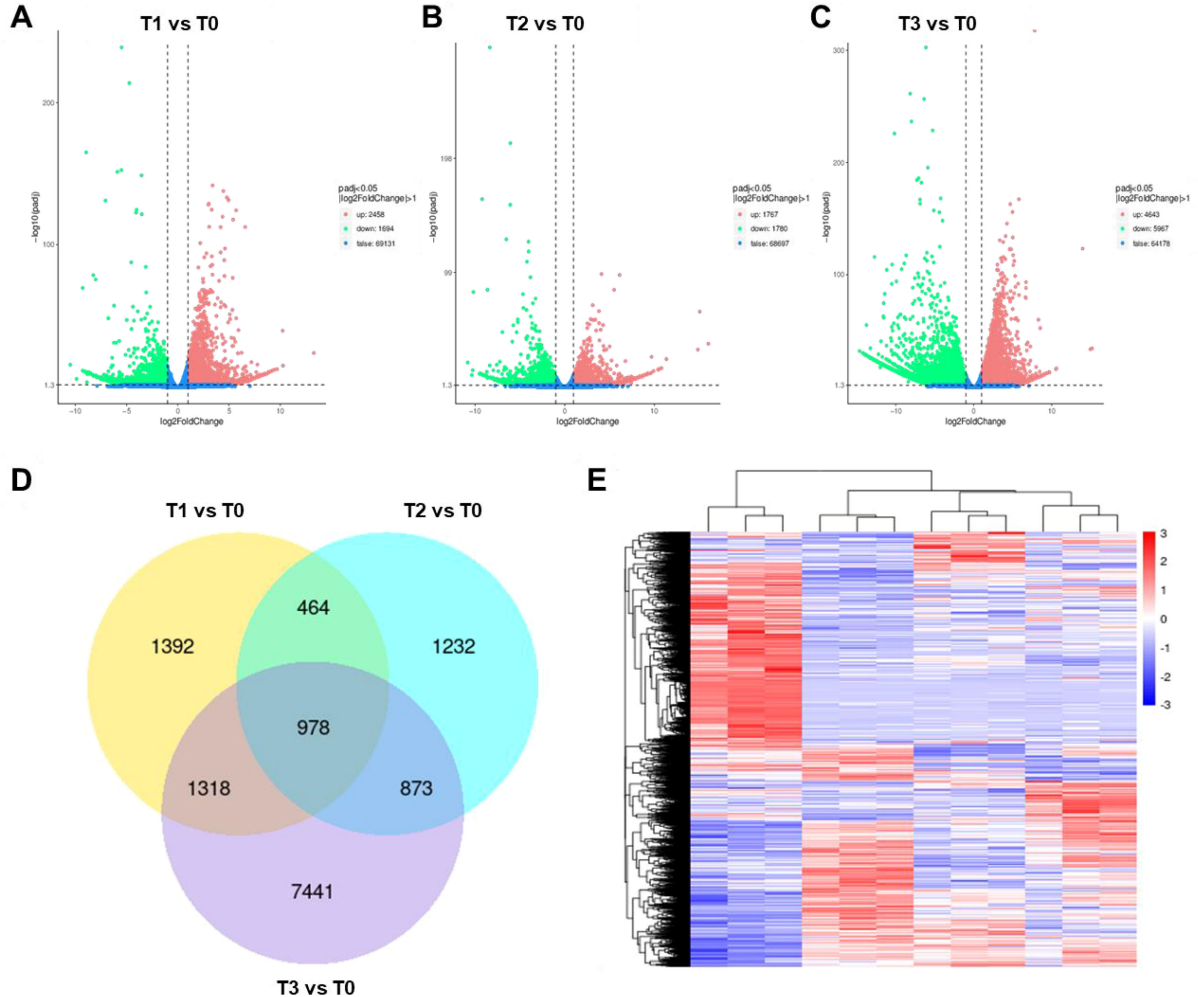
Transcriptomic analysis of *S. chinensis* under fertilization treatment. **(A–C)** Volcano plots displaying differentially expressed genes (DEGs) upon fertilization treatment (T1, T2, and T3, respectively) compared to the control (T0). **(D)** Venn diagram illustrating the overlap of DEGs among the different treatment groups. **(E)** Heatmap showing the expression patterns of identified DEGs.

As shown in **Figure 1**, bio-organic fertilizer significantly influenced both the yield and quality of *S. chinensis*. To further investigate the mechanisms underlying these improvements, fruit samples from plants treated with different levels of bio-organic fertilizer were collected and subjected to transcriptome sequencing. First, the quality of the RNA-seq samples was assessed. Global evaluation confirmed that all samples were suitable for sequencing analysis (**Supplementary Figures S1** and S2). We then examined the gene expression profiles of *S. chinensis* under different fertilizer treatments. As illustrated in **Figures 2A–C**, the application of bio-organic fertilizer markedly altered the expression patterns of numerous genes. Differential gene expression analysis revealed 1,392 DEGs in T1 vs. T0, 1,232 DEGs in T2 vs. T0, and 7,441 DEGs in T3 vs. T0. Among these, 978 DEGs associated with plant growth and nutrient accumulation were commonly identified across all three comparison groups (**Figure 2D**).

To visualize these expression changes, a heatmap was generated, displaying distinct up- and downregulation patterns of DEGs in response to different fertilizer treatments (**Figure 2E**). Further classification showed that in T1 vs. T0, 891 genes were downregulated; in T2 vs. T0, 1,017 genes were downregulated; and in T3 vs. T0, 5,091 genes were downregulated. A total of 238 downregulated genes were common to all three groups (**Supplementary Figure S2**). Similarly, analysis of upregulated genes identified 720 in T1 vs. T0, 436 in T2 vs. T0, and 2,661 in T3 vs. T0, with 641 upregulated genes shared across all groups (**Supplementary Figure S5**).

### Transcriptome analysis of *S. chinensis* under different bio-organic fertilizer regimens

**Figure 3:**
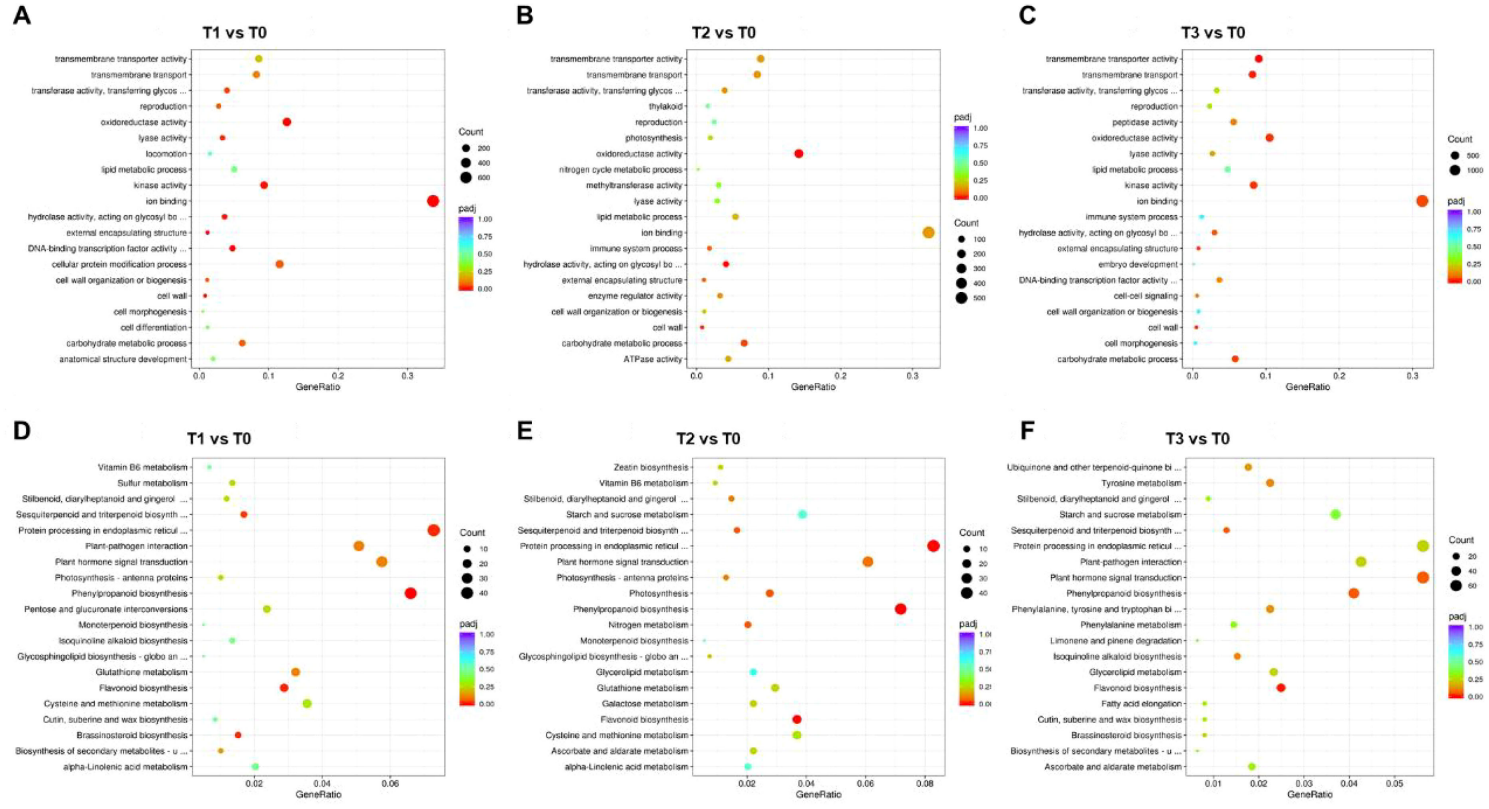
Functional enrichment analysis of DEGs in *S. chinensis* under fertilization. **(A–C)** GO enrichment analysis of DEGs identified from comparisons T1 vs. T0, T2 vs. T0, and T3 vs. T0, respectively. **(D–F)** KEGG enrichment analysis of DEGs from the same comparative groups, respectively.

To further investigate the functional roles of DEGs in *S. chinensis* under different bio-organic fertilizer treatments, we performed GO and KEGG enrichment analyses. In the comparison T1 vs. T0, DEGs were significantly enriched in GO terms including oxidoreductase activity, lyase activity, kinase activity, ion binding, hydrolase activity, external encapsulating structure, DNA-binding transcription factor activity, cellular protein modification process, and carbohydrate metabolic process (**Figure 3A**). For T2 vs. T0, enriched GO terms included oxidoreductase activity, hydrolase activity, and carbohydrate metabolic process (**Figure 3B**). In T3 vs. T0, DEGs were associated with transmembrane transporter activity, oxidoreductase activity, kinase activity, ion binding, hydrolase activity, and carbohydrate metabolic process (**Figure 3C**). We further analyzed the functional enrichment of down- and upregulated genes separately. In T1 vs. T0, downregulated genes were enriched in oxidoreductase activity, external encapsulating structure, and cell wall (**Supplementary Figure S4A**). In T2 vs. T0, downregulated genes were involved in oxidoreductase activity, ion binding, hydrolase activity, external encapsulating structure, and carbohydrate metabolic process (**Supplementary Figure S4B**). In T3 vs. T0, downregulated genes were related to peptidase activity and DNA binding (**Supplementary Figure S4C**). Among upregulated genes in T1 vs. T0, enrichment was observed in transferase activity, reproduction, oxidoreductase activity, lyase activity, kinase activity, DNA binding transcription factor activity, and cellular protein modification process (**Supplementary Figure S6A**). In T2 vs. T0, upregulated genes were associated with transferase activity, thylakoid, photosynthesis, oxidoreductase activity, and lyase activity (**Supplementary Figure S6B**). In T3 vs. T0, upregulated genes were involved in transmembrane transporter activity, oxidoreductase activity, lyase activity, lipid metabolic process, kinase activity, ion binding, hydrolase activity, cellular protein modification process, and carbohydrate metabolic process (**Supplementary Figure S6C**).

KEGG pathway analysis revealed that in T1 vs. T0, DEGs were enriched in sesquiterpenoid and triterpenoid biosynthesis, protein processing in endoplasmic reticulum, phenylpropanoid biosynthesis, flavonoid biosynthesis, and brassinosteroid biosynthesis (**Figure 3D**). In T2 vs. T0, DEGs were related to sesquiterpenoid and triterpenoid biosynthesis, protein processing, plant hormone signal transduction, photosynthesis, phenylpropanoid biosynthesis, and flavonoid biosynthesis (**Figure 3E**). In T3 vs. T0, DEGs were involved in plant hormone signal transduction, photosynthesis, phenylpropanoid biosynthesis, and flavonoid biosynthesis (**Figure 3F**). Additionally, KEGG enrichment was conducted for down- and upregulated genes separately. In T1 vs. T0, downregulated genes were enriched in protein processing, photosynthesis, phenylpropanoid biosynthesis, and DNA replication (**Supplementary Figure S4D**). In T2 vs. T0, downregulated genes were involved in protein processing, photosynthesis, phenylpropanoid biosynthesis, and flavonoid biosynthesis (**Supplementary Figure S4E**). In T3 vs. T0, downregulated genes were associated with spliceosome, protein processing, plant hormone signal transduction, and mismatch repair (**Supplementary Figure S4F**). Among upregulated genes in T1 vs. T0, enrichment was observed in sesquiterpenoid and triterpenoid biosynthesis, plant hormone signal transduction, phenylpropanoid biosynthesis, flavonoid biosynthesis, and brassinosteroid biosynthesis (**Supplementary Figure S6D**). In T2 vs. T0, upregulated genes were related to sesquiterpenoid and triterpenoid biosynthesis, plant hormone signal transduction, photosynthesis, and flavonoid biosynthesis (**Supplementary Figure S6E**). In T3 vs. T0, upregulated genes were involved in tyrosine metabolism, photosynthesis, phenylpropanoid biosynthesis, and flavonoid biosynthesis (**Supplementary Figure S6F**). Furthermore, to elucidate the molecular mechanisms underlying the fertilization effects, we conducted a weighted gene co-expression network analysis (WGCNA) on *S. chinensis* transcriptome data (**Supplementary Figure S7**).

### Metabolome sequencing of *S. chinensis* under different bio-organic fertilizer treatments

**Figure 4:**
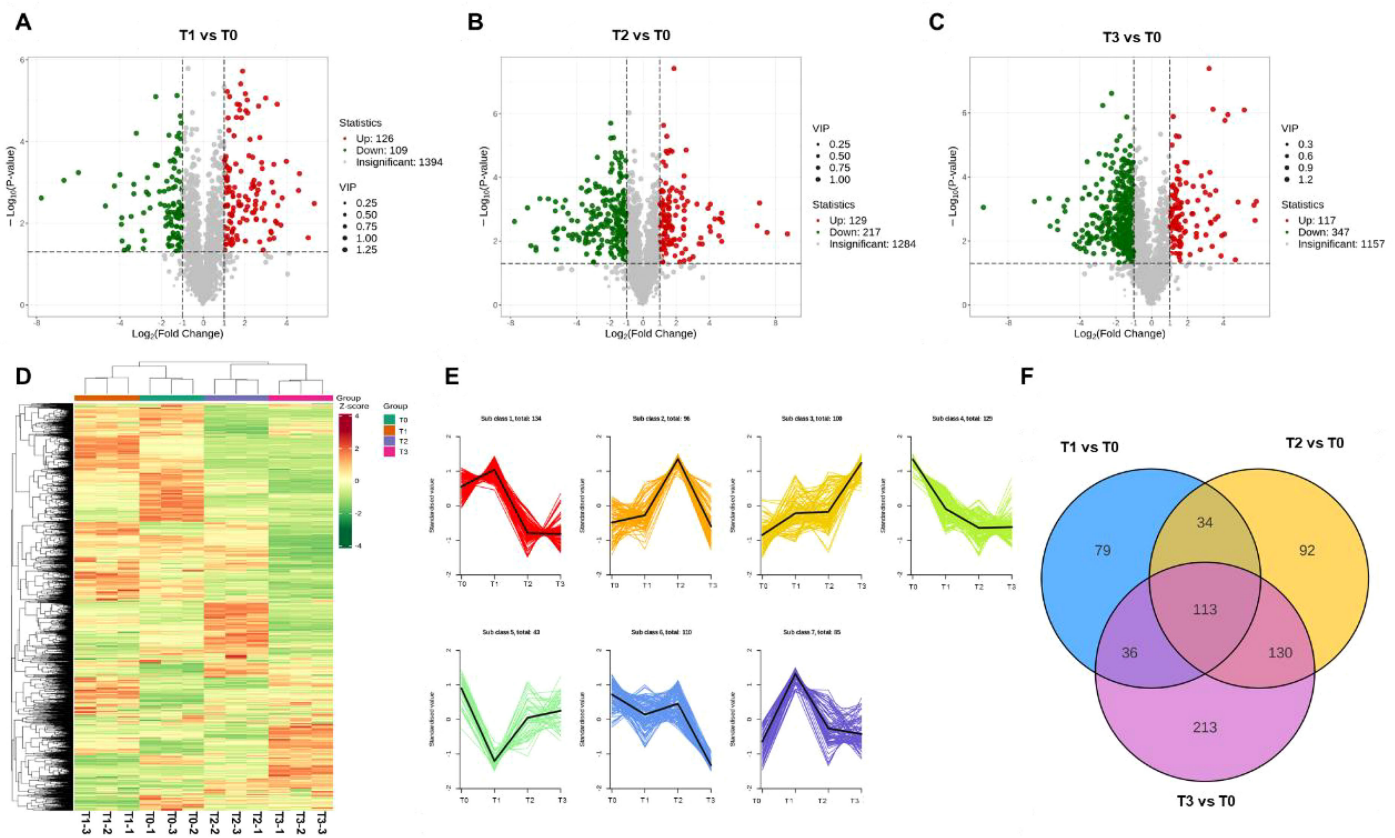
Metabolomic profiling of *S. chinensis* under fertilization treatment. **(A–C)** Volcano plots of differentially accumulated metabolites (DAMs) in comparisons T1 vs. T0, T2 vs. T0, and T3 vs. T0, respectively. **(D)** Heatmap displaying DAMs across different fertilization conditions. **(E)** k-means clustering analysis of metabolite profiles. The metabolites are grouped into seven clusters, with the number of metabolites in each cluster indicated. The x-axis represents different fertilization treatments, and the y-axis represents the Z-score of metabolite abundance. The colored lines depict the accumulation dynamics of individual metabolites, while the black line in each cluster shows the representative trend. **(F)** Venn diagram illustrating the overlap of DAMs identified under different fertilization treatments.

Bio-organic fertilizer influenced both the yield and quality of *S. chinensis* (**Figure 1**). To further investigate the metabolomic profiles of *S. chinensis* fruits under different bio-organic fertilizer treatments, we collected fruit samples from each fertilizer group and conducted metabolome sequencing. First, we assessed the quality of the sequencing samples. Global evaluation of the metabolome data, including PCA, demonstrated acceptable intrinsic biological variation among samples, indicating their suitability for subsequent analysis (**Supplementary Figure S8**). We then analyzed the untargeted metabolomic profiles of *S. chinensis* under different fertilizer conditions. As shown in **Figures 4A–C**, the metabolite profiles of fruits varied considerably across treatment groups. Specifically, in the comparison T1 vs. T0, 126 metabolites were upregulated and 109 were downregulated; 129 were upregulated and 217 downregulated in T2 vs. T0; and 117 were upregulated and 347 downregulated in T3 vs. T0. Based on these differential metabolites, we generated a heatmap to visualize their expression patterns (**Supplementary Figure S8**). We also examined metabolite abundances across groups (**Figure 4D**) and performed clustering analysis, which categorized the abundance patterns of metabolites in the four groups into seven distinct clusters (**Figure 4E**). Furthermore, we statistically analyzed the number of differential metabolites unique to each comparison. There were 79 differential metabolites specific to T1 vs. T0, 92 to T2 vs. T0, and 213 to T3 vs. T0. In total, 113 metabolites were common to all three treatment groups (**Figure 4F**). These shared metabolites are primarily associated with plant growth and nutrient accumulation.

### Metabolomic profiling of *S. chinensis* in response to varying bio-organic fertilizer regimens

**Figure 5:**
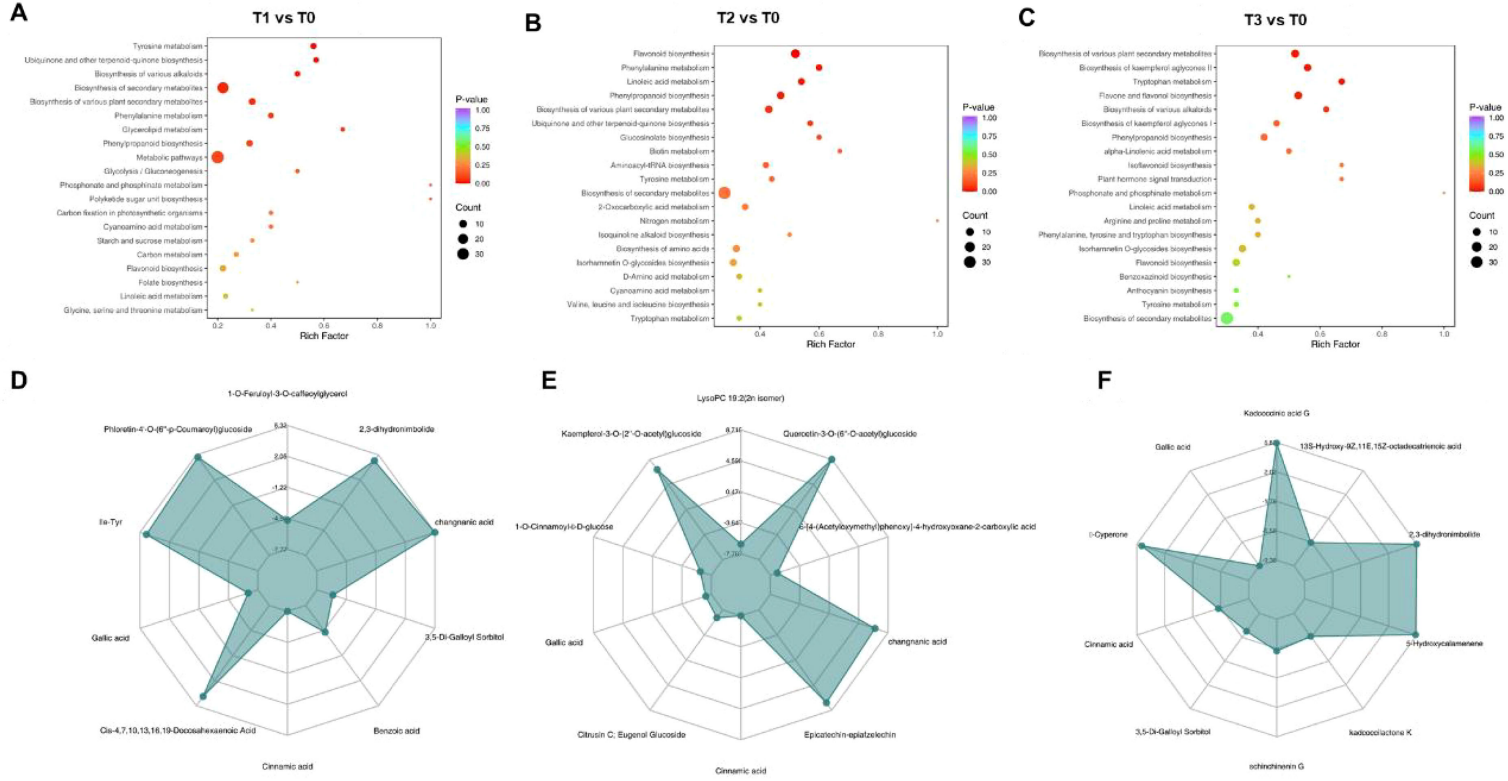
Analysis of metabolomic changes in *S. chinensis* under different fertilization regimes. (**A**–**C**) KEGG pathway enrichment analysis of differentially accumulated metabolites (DAMs) in T1, T2, and T3 compared to T0, respectively. (**D**–**F**) Radar charts visualizing the accumulation patterns of DAMs in T1, T2, and T3 versus T0, respectively.

**Figure 6:**
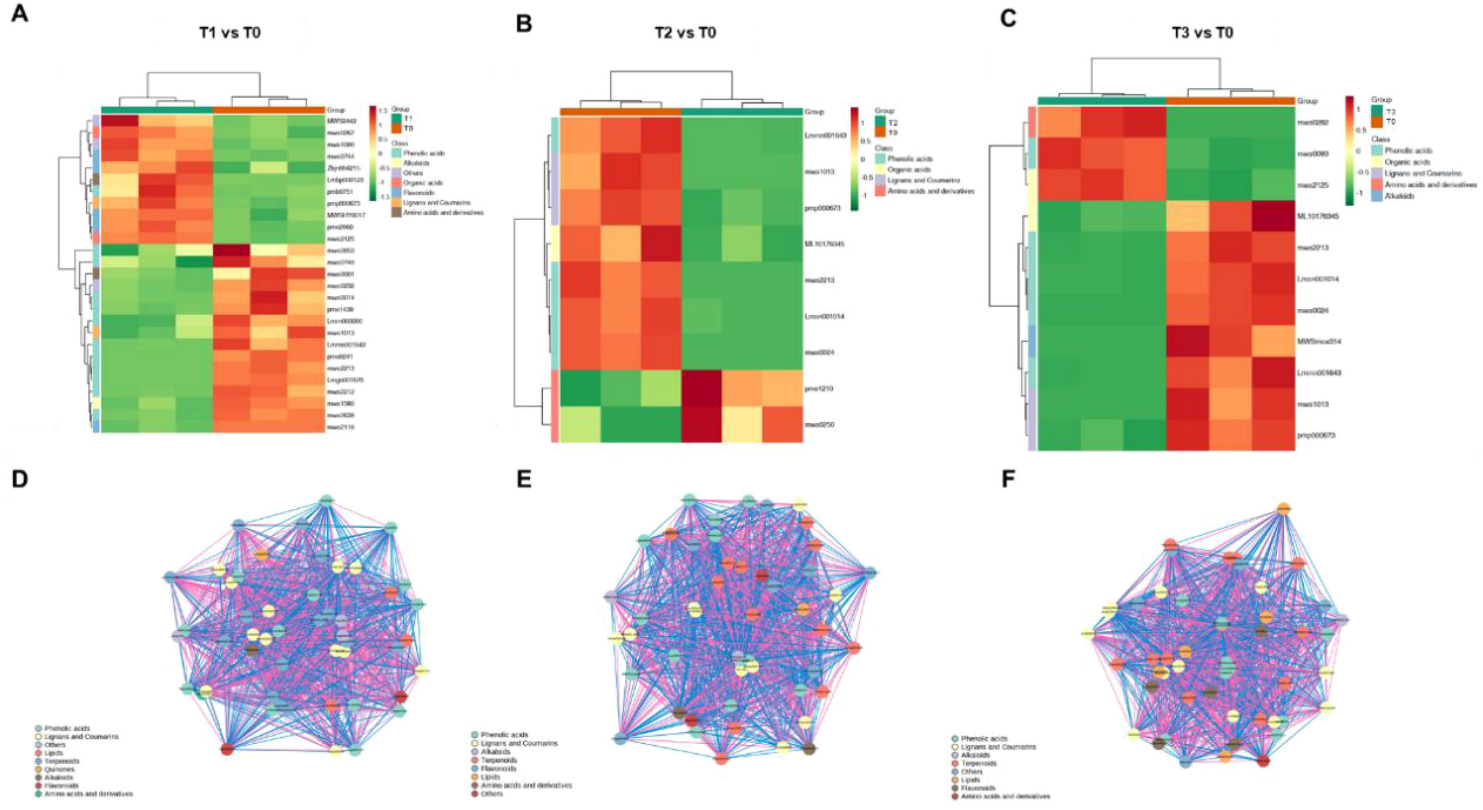
Analysis of DAMs in *S. chinensis* under fertilization. **(A–C)** Heatmaps displaying the abundance patterns of DAMs identified in the comparisons of T1 vs. T0, T2 vs. T0, and T3 vs. T0, respectively. **(D–F)** Correlation analysis of DAMs in T1 vs. T0, T2 vs. T0, and T3 vs. T0, respectively.

To further investigate the functions of differentially accumulated metabolites in *S. chinensis* under different bio-organic fertilizer treatments, we performed KEGG pathway enrichment analysis. As shown in **Figures 5A–C**, the differentially accumulated metabolites in T1 vs. T0, T2 vs. T0, and T3 vs. T0 were primarily associated with flavone and flavonol biosynthesis, flavonoid biosynthesis, and the biosynthesis of various plant secondary metabolite pathways known to be involved in plant growth and nutrient accumulation. Additionally, we generated radar charts to visualize the accumulation patterns of metabolites across the different treatment groups (**Figures 5D–F**), which illustrate both the presence and relative abundance of key metabolites. These charts also help identify specific chemical compounds that may play important roles in each group. We further analyzed the enrichment pathways of the differentially accumulated metabolites. **Figures 6A–C** display the up- and downregulation of relevant metabolic pathways in each comparison group (T1 vs. T0, T2 vs. T0, and T3 vs. T0), which are functionally linked to plant growth and nutrient accumulation. Moreover, correlation analyses were conducted on the differentially accumulated metabolites (**Figures 6D–F**; **Supplementary Figures S9** and S10). These metabolites were identified as phenolic acids, organic acids, flavonoids, lignans and coumarins, amino acids and their derivatives, and alkaloids across the three comparison groups. In summary, the altered metabolic pathways are closely associated with plant growth and nutrient accumulation, consistent with the observed phenotypic changes in plant growth and nutrient content. Furthermore, an integrated analysis of the transcriptome and metabolome was conducted to identify key regulatory relationships underlying the observed phenotypic changes (**Supplementary Figure S11**).

## Discussion

*S. chinensis*, a well-known traditional Chinese medicine, belongs to the *Magnoliaceae* family. Extensive research has been conducted to explore various aspects of this plant, including its antioxidant and antimicrobial activities, polyphenolic composition, and medicinal potential. Previous studies have qualitatively and quantitatively evaluated the antioxidant and antimicrobial properties of its leaves and fruits, highlighting the abundance of polyphenolic compounds (Mocan et al., 2014b). The anti-inflammatory effects of *S. chinensis* fruit extract had also been investigated in murine macrophages, with a focus on the inactivation of the nuclear factor-κB and mitogen-activated protein kinase signaling pathways (Chen et al., 2014). Additionally, schisandrin B and deoxyschizandrin from *S. chinensis* have been identified as potential scaffolds to develop novel HIV-1 reverse transcriptase inhibitors (Xu et al., 2015). Schisantherin A has been shown to protect against dopaminergic neuron damage and cytotoxicity, likely through the modulation of the ROS/NO and AKT/GSK3β pathways (Zhang et al., 2015). A comprehensive review further summarized the bioactive components, pharmacological properties, and medicinal potential of *S. chinensis*, emphasizing its beneficial effects in biological systems (Szopa et al., 2017). Enzymatic assays and molecular modeling studies on its lignans and phenolics have revealed their enzyme inhibitory potential and bioactivity (Mocan et al., 2016). Moreover, the ethanol extract of *S. chinensis* fructus was demonstrated to ameliorate experimentally induced atherosclerosis in rats by enhancing antioxidant capacity and improving endothelial dysfunction (Chen et al., 2018). Finally, a review of its main lignan components highlighted their effects on cytochrome P450 and P-glycoprotein activities, suggesting a potential for drug–drug interactions (Zhang et al., 2022). Collectively, these studies underscore the diverse pharmacological properties of *S. chinensis*, including antioxidant, anti-inflammatory, and neuroprotective effects, positioning it as a promising candidate for further development in natural medicine.

Nutrient absorption and plant growth are highly dependent on nutrient availability in the soil solution. Maintaining optimal nutrient levels through appropriate fertilization is essential in nurseries and greenhouses (Wright, 1986), and improving nutrient use efficiency has become a central goal in plant nutrition management (Baligar et al., 2001). Field studies have shown that microbial inoculants, such as plant growth-promoting rhizobacteria (PGPR) and arbuscular mycorrhizal fungi (AMF), can significantly enhance nutrient uptake and plant growth within integrated nutrient management systems (Adesemoye et al., 2008)—for example, pseudomonads have been shown to improve wheat growth, yield, and nutrient use efficiency in a fertilizer-dependent manner, supporting their combined application with optimized fertilization to reduce chemical input (Shaharoona et al., 2008). Organic amendments like vermicompost have also gained attention for their role in promoting plant growth and serving as effective soil conditioners and biocontrol agents, offering a sustainable alternative to synthetic fertilizers (Hayat et al., 2010) (Joshi et al., 2015). Additionally, biostimulants and biochar have been explored for their ability to improve nutrient uptake and soil fertility (Halpern et al., 2015; Ding et al., 2016). Overall, these approaches highlight the importance of sustainable nutrient management strategies to enhance plant growth and nutrient utilization in agriculture. Extensive research has demonstrated that organic fertilizers substantially alter soil nutrient availability and plant physiology, thereby enhancing plant growth, nutrient uptake, and the synthesis of secondary metabolites. This underscores their critical role in sustainable agricultural practices. Beyond direct nutrition, organic fertilizers enhance soil health and nutrient dynamics by modifying microbial populations and improving physical properties. Supporting this, Aziz demonstrated that amending biogas slurry with zinc oxide nanoparticles and zeolite increased the soil’s mineral nitrogen content, a key indicator of enhanced nutrient availability for plants (Aziz et al., 2019). Organic fertilizers can bolster plant resilience and secondary metabolite production by modulating nutrient availability and root-associated microbial activity. In our study, the application of bio-organic fertilizer improved the fruit shape index and yield of *S. chinensis*, along with increased content of lignans, flavonoids, polysaccharides, and polyphenols (**Figure 1**). The T2 treatment group, which received 100 kg/667 m² of bio-organic fertilizer, 35 kg/667 m² of 13-8–9 organic–inorganic compound fertilizer, 35 kg/667 m² of 15-6–24 compound fertilizer, and 17.5 kg/667 m² of 8-5–38 compound fertilizer, exhibited the best performance.

In recent years, multi-omics approaches have been widely adopted to study plant responses to environmental changes—for instance, melatonin was found to alter the secondary metabolite profile in grape berry skin by promoting *VvMYB14*-mediated ethylene biosynthesis (Ma et al., 2021). Multi-omics analyses have also been applied to study perchlorate stress in *Chlamydomonas reinhardtii*, autotoxic ginsenoside-induced recruitment of beneficial *Burkholderia* strain B36 (Zhang et al., 2023), cold tolerance in citrus involving sphingosine and chlorogenic acid (Xiao et al., 2024), and preharvest browning in apple peel (Wang et al., 2023; Wang et al., 2024). These studies demonstrate the power of integrated omics in elucidating molecular mechanisms underlying stress responses and metabolic regulation (Meng et al., 2022). In our research, we employed transcriptomic and metabolomic analyses to investigate how fertilization enhances the yield and quality of *S. chinensis*. We identified key gene network modules associated with plant growth and nutrient metabolism (**Figures 2**–6).

Our study revealed that bio-organic fertilizer enhanced both growth and nutrient accumulation in *S. chinensis*, particularly under the T2 treatment. Transcriptomic and metabolomic analyses confirmed these phenotypic observations and highlighted specific genes and metabolites involved in these responses. Overall, this multi-omics study provides new insights into the molecular mechanisms through which fertilization enhances the growth and nutritional quality of *S. chinensis*, offering a genetic and metabolic perspective to optimize fertilization strategies.

## Data Availability

The datasets generated and analysed during the current study are available in the Figshare repository. All the raw sequencing data generated for this project have been deposited in the NCBI database with the number of PRJNA1283067.

## Supplementary material

Supplementary Figure 1 Global evaluation of the RNA-seq experiment. **(A)** PCA displaying the intrinsic biological variation among samples. **(B)** Pearson’s correlation coefficients of FPKM values between pairs of samples.

Supplementary Figure 2 qRT-PCR confirmed the quality of the transcriptome. **(A)** FPKM value of the genes. **(B)** qRT-PCR of genes.

Supplementary Figure 3 Downregulated gene profiling of *S. chinensis* under fertilization. **(A)** The Venn diagram showed downregulated genes under fertilization. **(B)** The heatmaps showed downregulated genes of *S. chinensis* under fertilization.

Supplementary Figure 4 Transcriptome of downregulated gene analysis in *S. chinensis* under fertilization. **(A–C)** GO enrichment analysis of downregulated genes of *S. chinensis* in T1 vs. T0, T2 vs. T0, and T3 vs. T0, respectively. **(D–F)** KEGG enrichment analysis of downregulated genes of *S. chinensis* in T1 vs. T0, T2 vs. T0, and T3 vs. T0, respectively.

Supplementary Figure 5 Upregulated gene profiling of *S. chinensis* under fertilization. **(A)** The Venn diagram showed upregulated genes under fertilization. **(B)** The heatmaps showed upregulated genes of *S. chinensis* under fertilization.

Supplementary Figure 6 Transcriptome of upregulated gene analysis in *S. chinensis* under fertilization. **(A–C)** GO enrichment analysis of upregulated genes of *S. chinensis* in T1 vs. T0, T2 vs. T0, and T3 vs. T0, respectively. **(D–F)** KEGG enrichment analysis of upregulated genes of *S. chinensis* in T1 vs. T0, T2 vs. T0, and T3 vs. T0, respectively.

Supplementary Figure 7 Weighted gene co-expression network analysis (WGCNA) in *S. chinensis* under fertilization. **(A)** Module hierarchical clustering tree. **(B)** Heatmap of inter-module correlation. **(C)** Heatmap of correlation between samples and modules. **(D)** Module gene clustering heatmap.

Supplementary Figure 8 Global evaluation of the metabolism experiment. **(A)** Pearson’s correlation coefficients between pairs of samples. **(B)** PCA displaying the intrinsic biological variation among samples.

Supplementary Figure 9 Effect on the metabolome of *S. chinensis* under different fertilization. **(A)** The heatmaps show differentially accumulated metabolites in T1 vs. T0. **(B)** The heatmaps show differentially accumulated metabolites in T2 vs. T0. **(C)** The heatmaps show differentially accumulated metabolites in T3 vs. T0.

Supplementary Figure 10 Metabolome of *S. chinensis* under different fertilization. **(A)** Correlation analysis of differentially accumulated metabolites in T1 vs. T0. **(B)** Correlation analysis of differentially accumulated metabolites in T2 vs. T0. **(C)** Correlation analysis of differentially accumulated metabolites in T3 vs. T0.

Supplementary Figure 11 Correlation between the transcriptome and metabolome in *S. chinensis* under fertilization. **(A–C)** Transcriptome and metabolome in *S. chinensis* in T1 vs. T0, T2 vs. T0, and T3 vs. T0, respectively.
