# Supplementary material for: Integrated transcriptomic and metabolomic analyses reveal the mechanisms underlying bio-organic fertilizer-mediated growth and nutrient enhancement in Schisandra chinensis (Turcz.) Baill
Source: Front Plant Sci. 2025 Nov 18;16:1662470. doi: 10.3389/fpls.2025.1662470 (PMC12670174; doi:10.3389/fpls.2025.1662470)
Supplement: Supplementary Figure 1 — Global evaluation of the RNA-seq experiment. (A) PCA displaying the intrinsic biological variation among samples. (B) Pearson’s correlation coefficients of FPKM values between pairs of samples. [file DataSheet1.pdf]

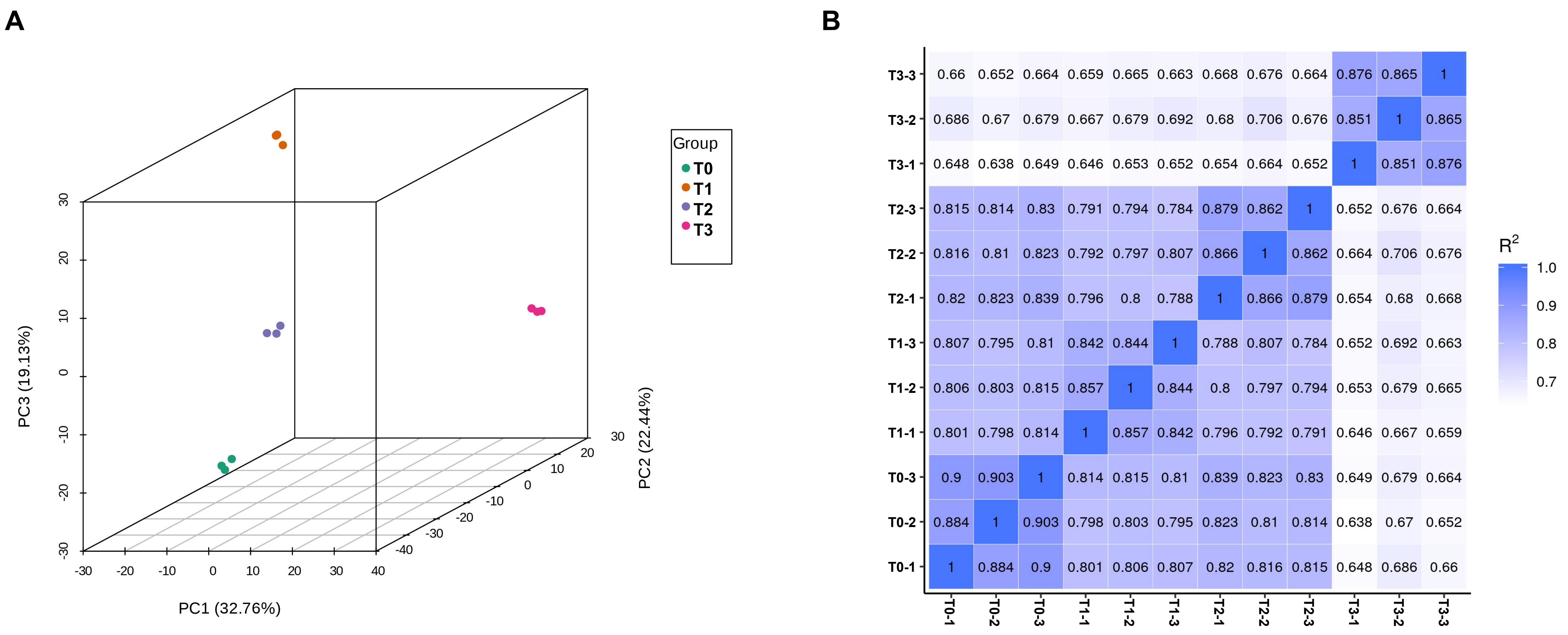

Supplement figure S1. Global evaluation of the RNA-seq experiment. A, PCA displaying the intrinsic biological variation among samples. **B,** Pearson's correlation coefficients of FPKM values between pairs of samples.

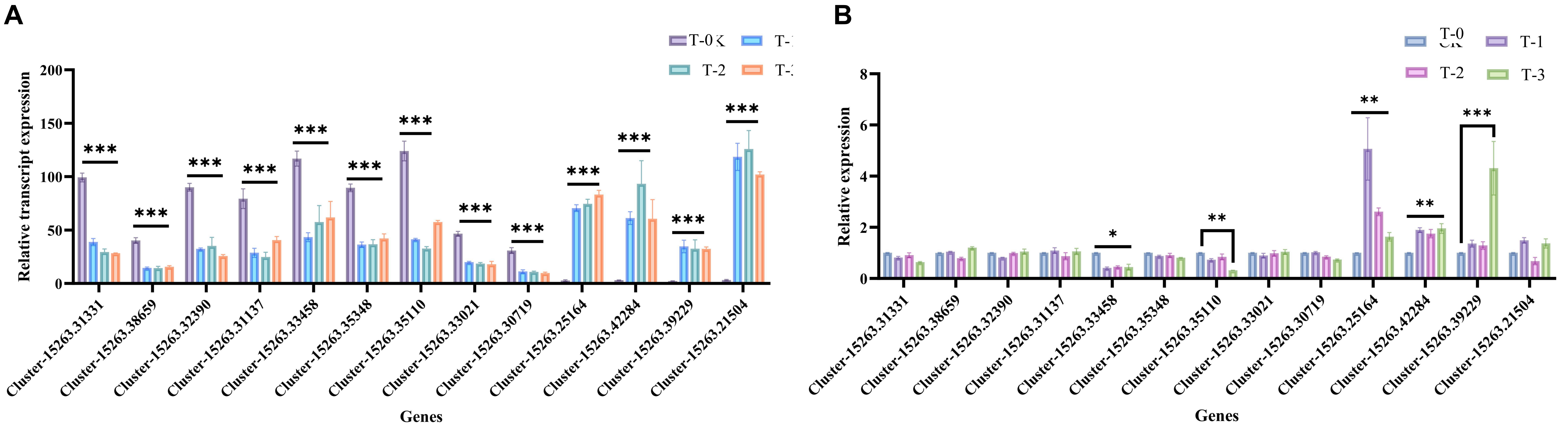

Supplement figure S2. qRT-PCR confirmed the quality of the transcriptome. A, FPKM value of the genes. **B,** qRT-PCR of genes.

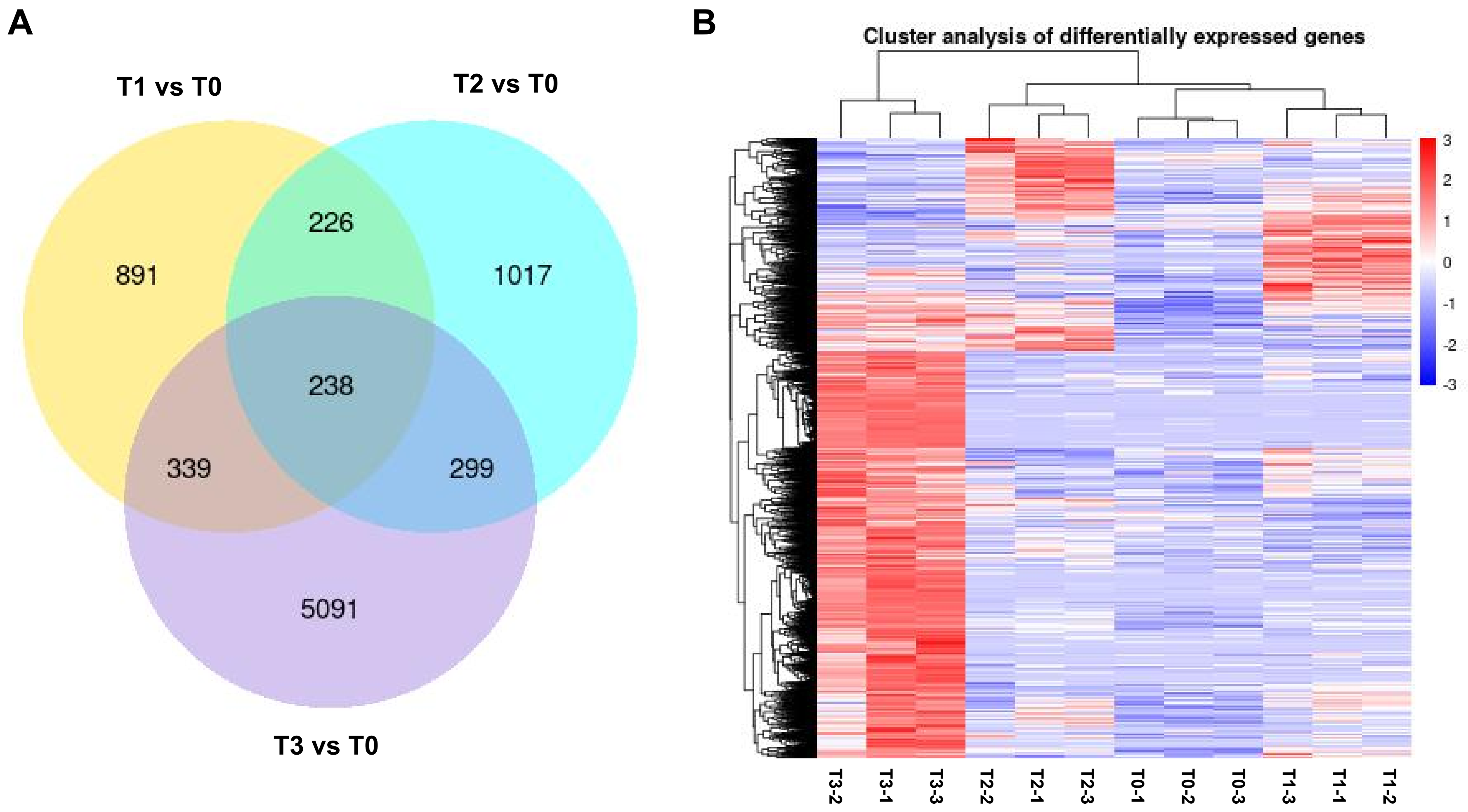

Supplement figure S3. Down-regulated gene profiling of *S. chinensis* under fertilization. A, The venn diagram showed down-regulated genes genes under fertilization. **B,** The heatmaps showed down-regulated genes of *S. chinensis* under fertilization.

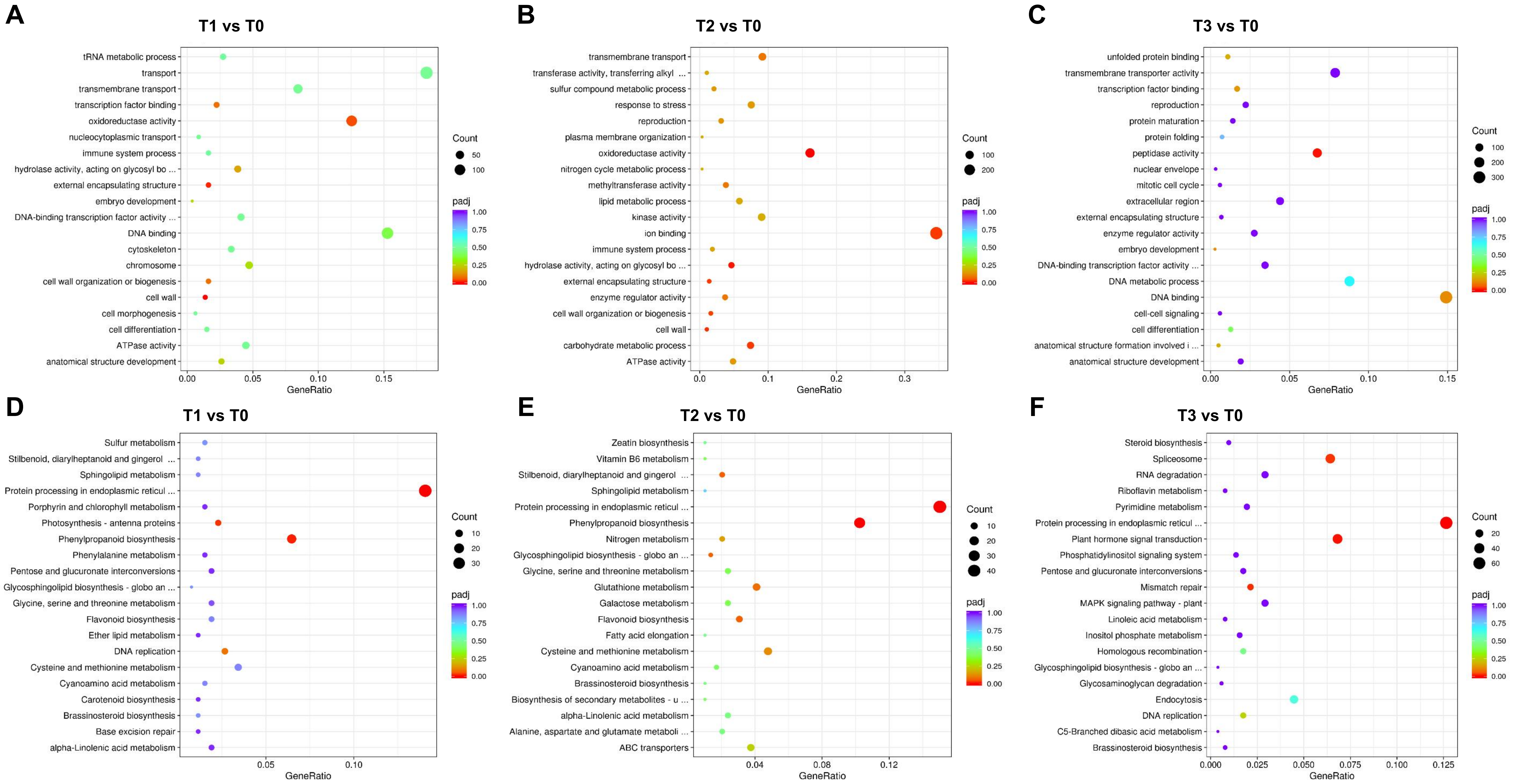

Supplement figure S4. Transcriptome of down-regulated genes analysis in *S. chinensis* under fertilization. A-C, GO enrichment analysis of down-regulated genes of *S. chinensis* inT1 vs T0, T2 vs T0, T3 vs T0. **D-F,** KEGG enrichment analysis of down-regulated genes of *S. chinensis* inT1 vs T0, T2 vs T0, T3 vs T0.

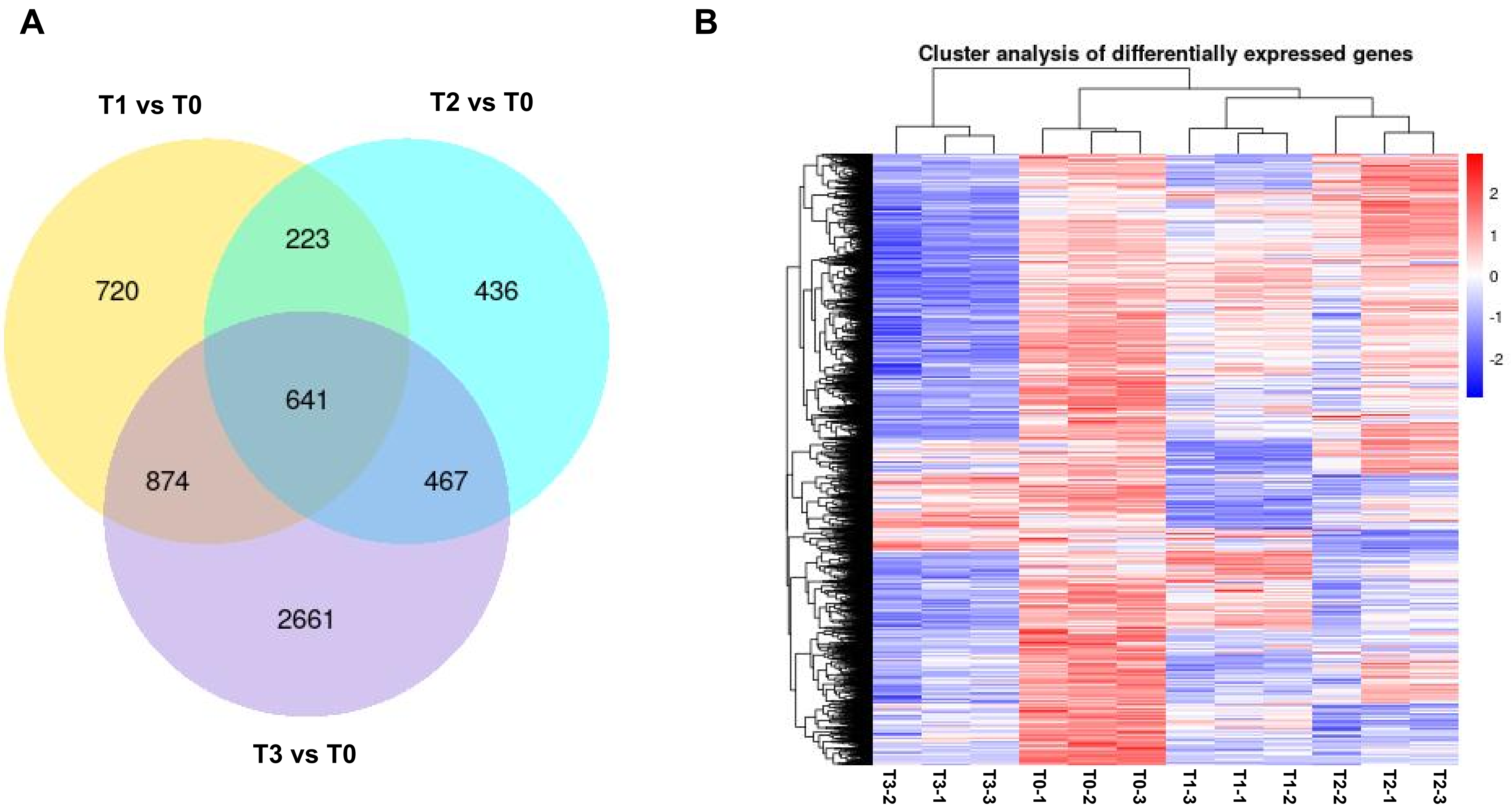

Supplement figure S5. Up-regulated gene profiling of *S. chinensis* under fertilization. A, The venn diagram showed up-regulated genes genes under fertilization. **B,** The heatmaps showed up-regulated genes of *S. chinensis* under fertilization.

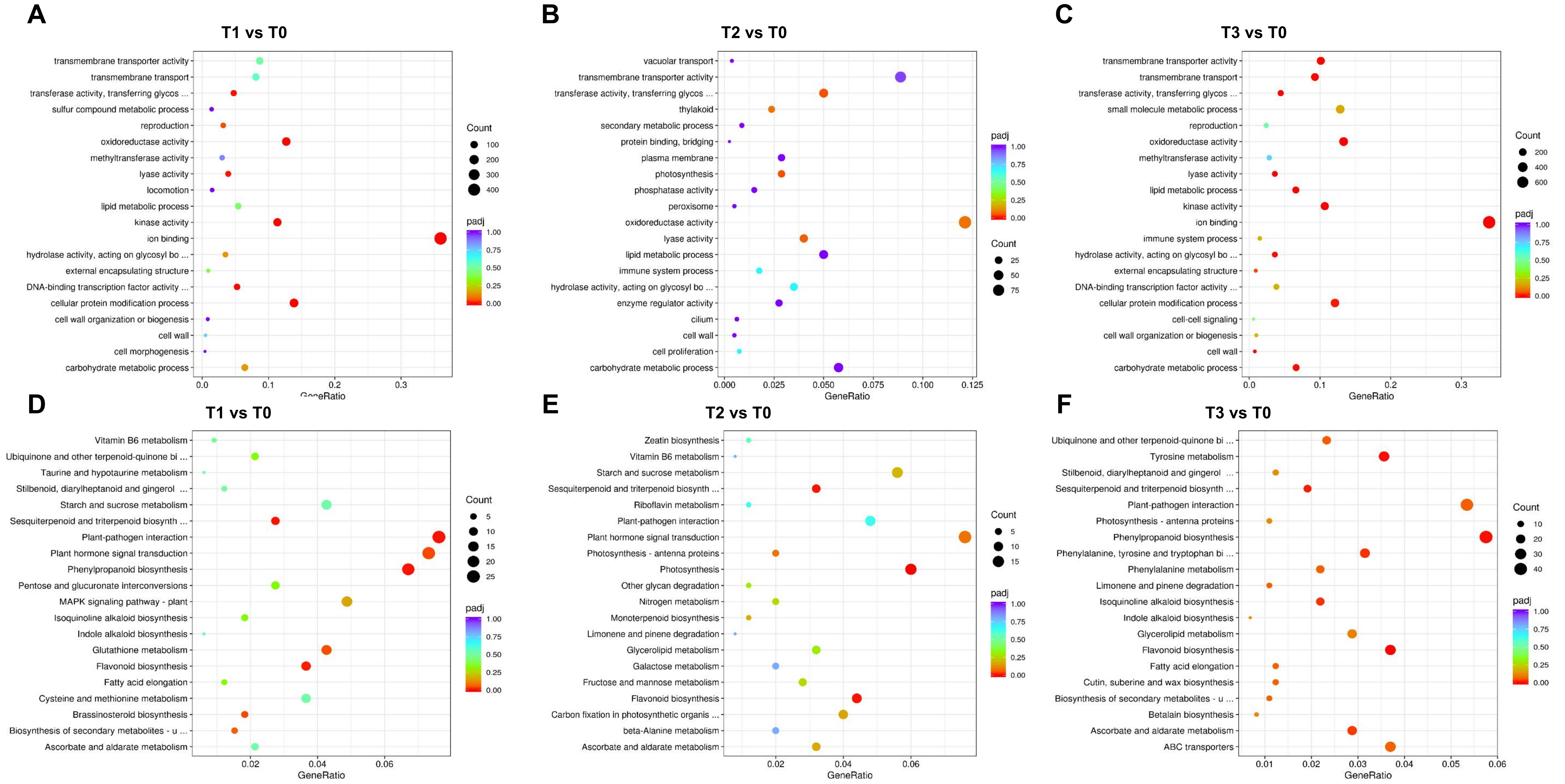

Supplement figure S6. Transcriptome of up-regulated genes analysis in *S. chinensis* under fertilization. A-C, GO enrichment analysis of up-regulated genes of *S. chinensis* inT1 vs T0, T2 vs T0, T3 vs T0. **D-F,** KEGG enrichment analysis of up-regulated genes of *S. chinensis* inT1 vs T0, T2 vs T0, T3 vs T0.

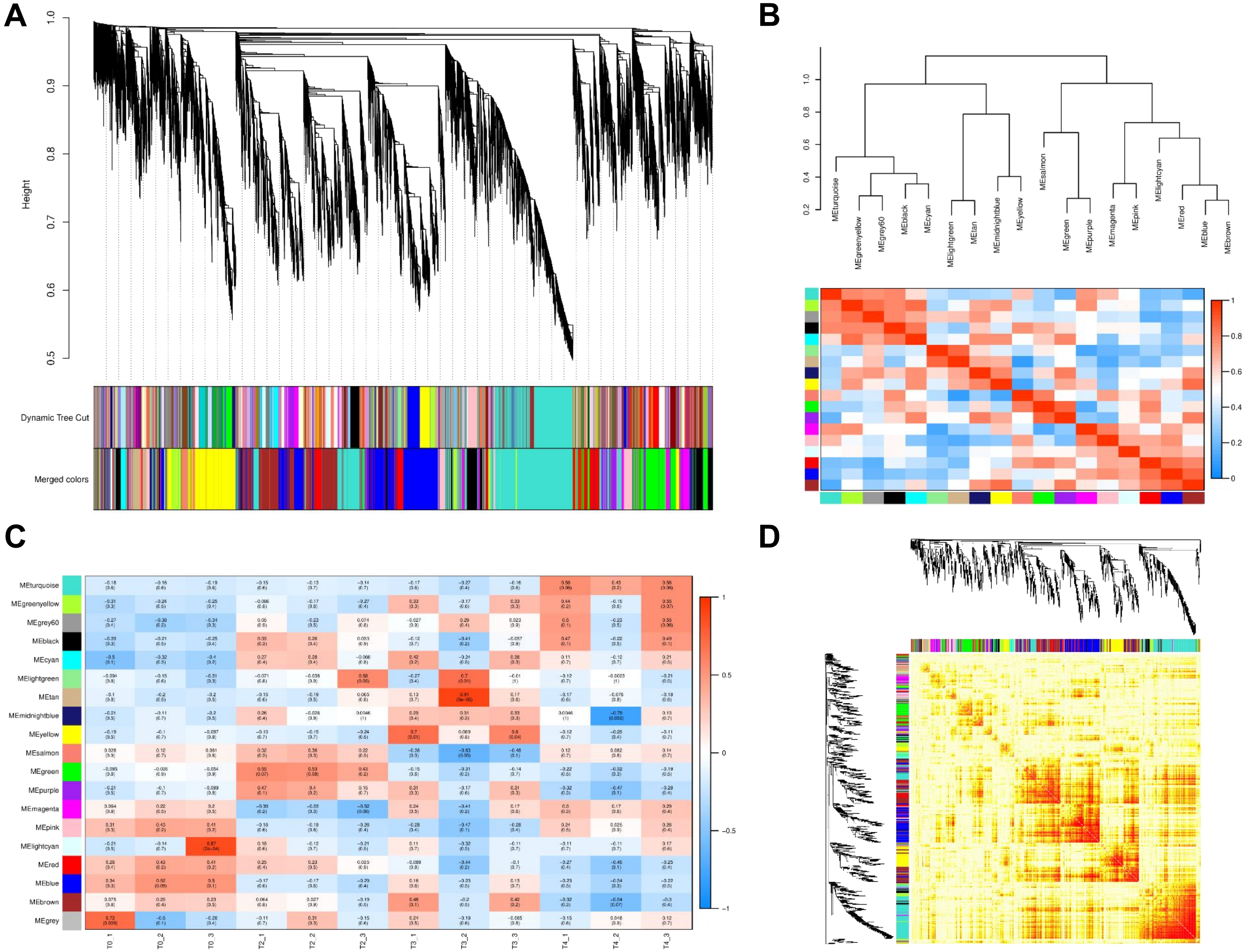

Supplement figure S7. Weighted Gene Co-expression Network Analysis (WGCNA) in *S. chinensis* under fertilization. A, Module hierarchical clustering tree. **B,** Heatmap of inter-module correlation. **C,** Heatmap of correlation between samples and modules. **D,** Module gene clustering heatmap.

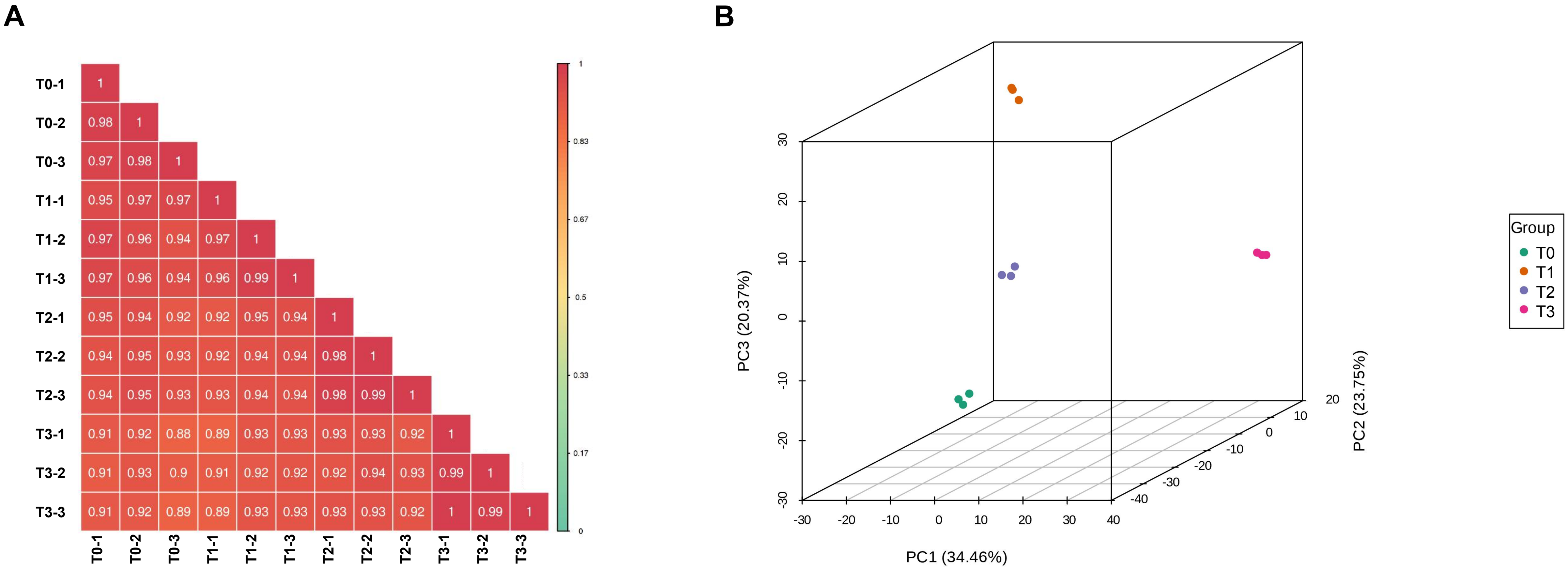

Supplement figure S8. Global evaluation of the metabolism experiment. A, Pearson's correlation coefficients between pairs of samples. **B,** PCA displaying the intrinsic biological variation among samples.

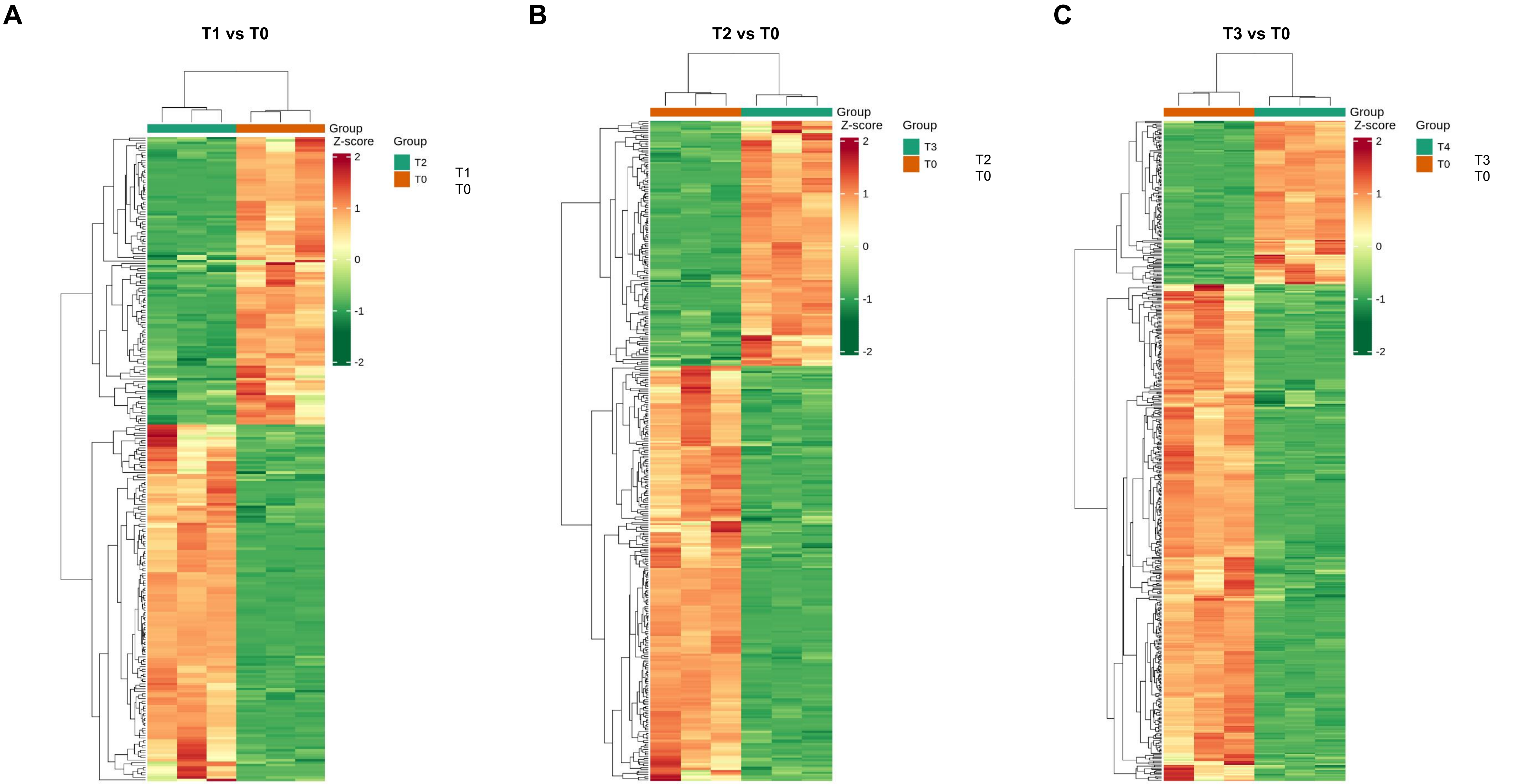

Supplement figure S9. Effect on the metabolome of *S. chinensis* under different fertilization. A, The heatmaps show differentially accumulated metabolites in T1 vs T0. **B,** The heatmaps show differentially accumulated metabolites in T2 vs T0. **C,** The heatmaps show differentially accumulated metabolites in T3 vs T0.

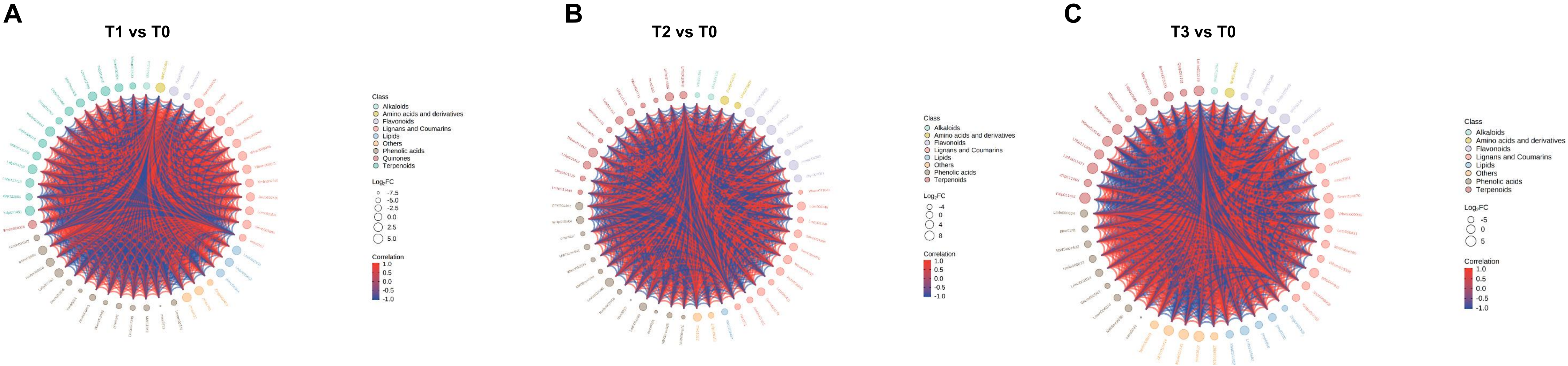

Supplement figure S10. Metabolome of *S. chinensis* under different fertilization. A, Correlation analysis of differentially accumulated metabolites in T1 vs T0. **B,** Correlation analysis of differentially accumulated metabolites in T2 vs T0. **C,** Correlation analysis of differentially accumulated metabolites in T3 vs T0.

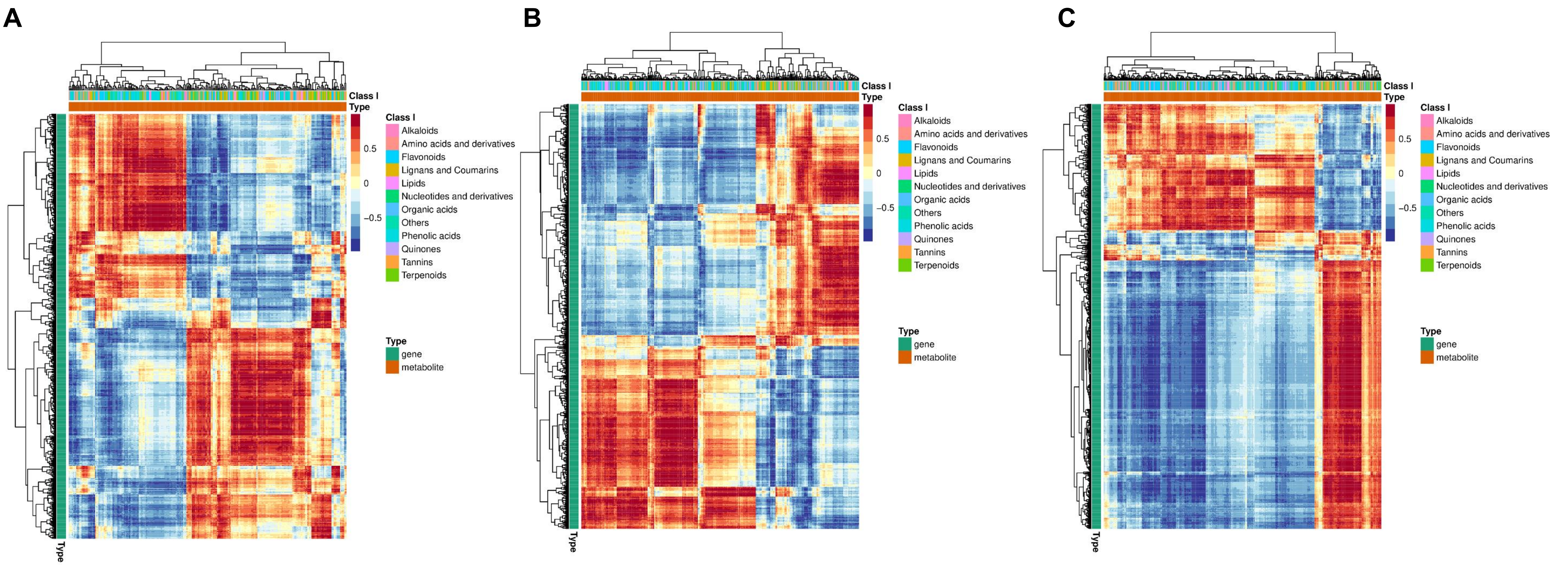

Supplement figure S11. The correlation between the transcriptome and metabolomein *S. chinensis* under fertilization. A-C, The transcriptome and metabolomein of *S. chinensis* inT1 vs T0, T2 vs T0, T3 vs T0.
