# Supplementary material for: Integrated transcriptomic and metabolomic analyses reveal the mechanisms underlying bio-organic fertilizer-mediated growth and nutrient enhancement in Schisandra chinensis (Turcz.) Baill
Source: Front Plant Sci. 2025 Nov 18;16:1662470. doi: 10.3389/fpls.2025.1662470 (PMC12670174; doi:10.3389/fpls.2025.1662470)
Supplement: Supplementary Figure 2 — qRT-PCR confirmed the quality of the transcriptome. (A) FPKM value of the genes. (B) qRT-PCR of genes. [file T1_vs_T0_common_KEGG_enrichment_bubble.pdf › Supplement data S1/T1_vs_T0/T1_vs_T0_common_KEGG_enrichment_bubble.pdf]

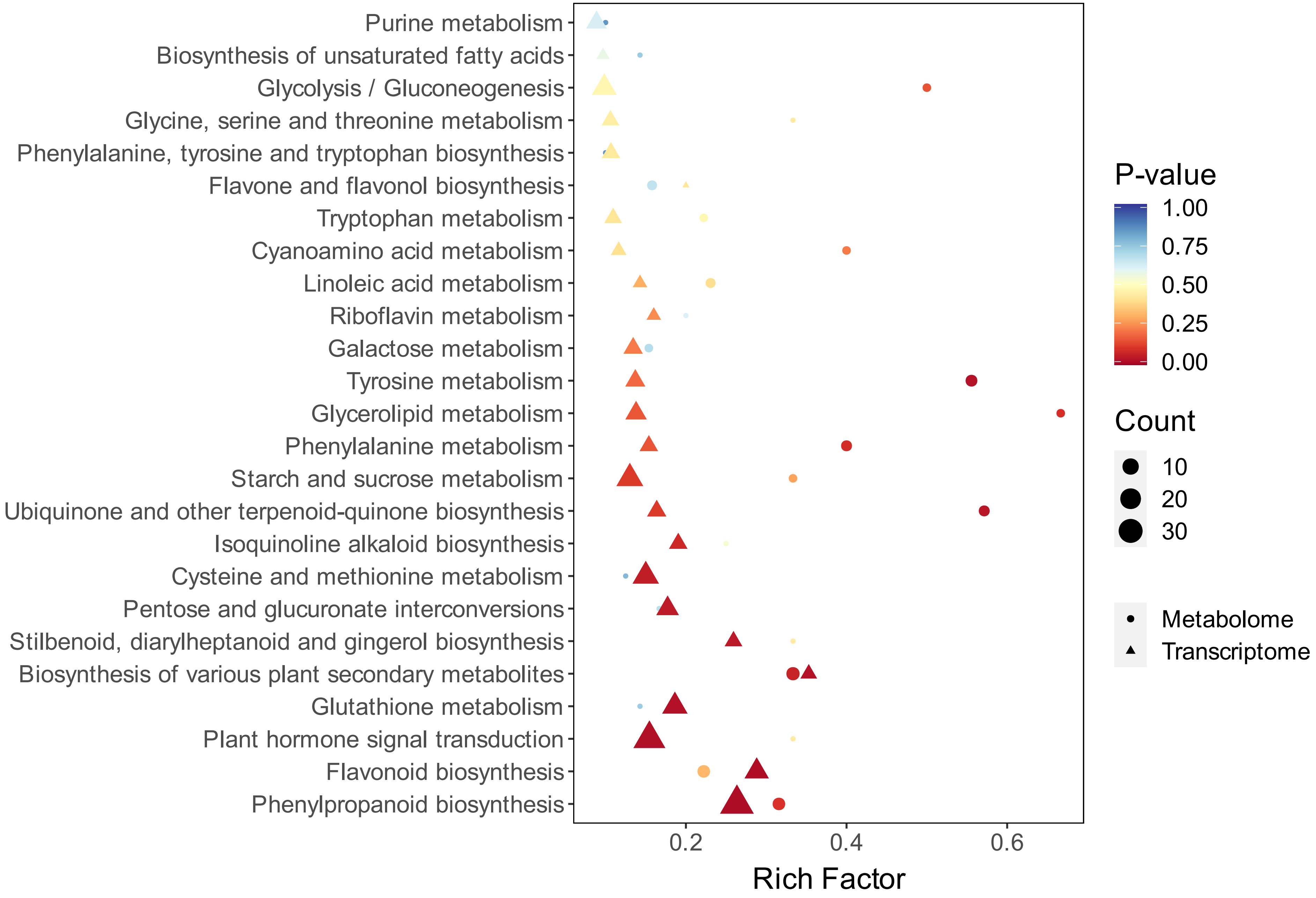
Purine metabolism
Biosynthesis of unsaturated fatty acids
Glycolysis / Gluconeogenesis
Glycine, serine and threonine metabolism
Phenylalanine, tyrosine and tryptophan biosynthesis
Flavone and flavonol biosynthesis
Tryptophan metabolism
Cyanoamino acid metabolism
Linoleic acid metabolism
Riboflavin metabolism
Galactose metabolism
Tyrosine metabolism
Glycerolipid metabolism
Phenylalanine metabolism
Starch and sucrose metabolism
Ubiquinone and other terpenoid-quinone biosynthesis
Isoquinoline alkaloid biosynthesis
Cysteine and methionine metabolism
Pentose and glucuronate interconversions
Stilbenoid, diarylheptanoid and gingerol biosynthesis
Biosynthesis of various plant secondary metabolites
Glutathione metabolism
Plant hormone signal transduction
Flavonoid biosynthesis
Phenylpropanoid biosynthesis
0.2
0.4
0.6
Rich Factor
P-value
1.00
0.75
0.50
0.25
0.00
Count
10
20
30
Metabolome
Transcriptome
