# Supplementary material for: Integrated transcriptomic and metabolomic analyses reveal the mechanisms underlying bio-organic fertilizer-mediated growth and nutrient enhancement in Schisandra chinensis (Turcz.) Baill
Source: Front Plant Sci. 2025 Nov 18;16:1662470. doi: 10.3389/fpls.2025.1662470 (PMC12670174; doi:10.3389/fpls.2025.1662470)
Supplement: Supplementary Figure 2 — qRT-PCR confirmed the quality of the transcriptome. (A) FPKM value of the genes. (B) qRT-PCR of genes. [file DataSheet2.zip › Supplement data S1/T3_vs_T0/maps/ko00940.html]

KEGG PATHWAY: Phenylpropanoid biosynthesis + Reference pathway


# Phenylpropanoid biosynthesis - Reference pathway


[
Pathway menu
|
Pathway entry
|
Image file
|
Help
]
